# EBNA1-Mediated Recruitment of a Histone H2B Deubiquitylating Complex to the Epstein-Barr Virus Latent Origin of DNA Replication

**DOI:** 10.1371/journal.ppat.1000624

**Published:** 2009-10-16

**Authors:** Feroz Sarkari, Teresa Sanchez-Alcaraz, Shan Wang, Melissa N. Holowaty, Yi Sheng, Lori Frappier

**Affiliations:** 1 Department of Molecular Genetics, University of Toronto, Toronto, Ontario, Canada; 2 Department of Biology, York University, Toronto, Ontario, Canada; Emory University, United States of America

## Abstract

The EBNA1 protein of Epstein-Barr virus (EBV) plays essential roles in enabling the replication and persistence of EBV genomes in latently infected cells and activating EBV latent gene expression, in all cases by binding to specific recognition sites in the latent origin of replication, *oriP*. Here we show that EBNA1 binding to its recognition sites *in vitro* is greatly stimulated by binding to the cellular deubiquitylating enzyme, USP7, and that USP7 can form a ternary complex with DNA-bound EBNA1. Consistent with the *in vitro* effects, the assembly of EBNA1 on *oriP* elements in human cells was decreased by USP7 silencing, whereas assembly of an EBNA1 mutant defective in USP7 binding was unaffected. USP7 affinity column profiling identified a complex between USP7 and human GMP synthetase (GMPS), which was shown to stimulate the ability of USP7 to cleave monoubiquitin from histone H2B *in vitro*. Accordingly, silencing of USP7 in human cells resulted in a consistent increase in the level of monoubquitylated H2B. The USP7-GMPS complex formed a quaternary complex with DNA-bound EBNA1 *in vitro* and, in EBV infected cells, was preferentially detected at the *oriP* functional element, FR, along with EBNA1. Down-regulation of USP7 reduced the level of GMPS at the FR, increased the level of monoubiquitylated H2B in this region of the origin and decreased the ability of EBNA1, but not an EBNA1 USP7-binding mutant, to activate transcription from the FR. The results indicate that USP7 can stimulate EBNA1-DNA interactions and that EBNA1 can alter histone modification at *oriP* through recruitment of USP7.

## Author Summary

Epstein-Barr virus (EBV) infections persist for the lifetime of the host largely due to the actions of the EBNA1 viral protein. EBNA1 enables the replication and stable persistence of EBV genomes and activates the expression of other EBV genes by binding to specific DNA sequences in the EBV genome. We have shown that the cellular protein USP7 stimulates EBNA1 binding to its DNA sequences and that EBNA1 recruits USP7 to the EBV genome, which in turn recruits another cellular protein GMP synthetase. The complex of USP7 and GMP synthetase then functions to alter the chromatin structure at a region of the EBV genome that controls EBV persistence. These changes to the EBV genome are likely important for enabling the persistence of EBV genomes in infected cells.

## Introduction

Epstein-Barr virus (EBV) is a gamma herpesvirus that infects over ninety percent of people worldwide. As part of its latent life cycle, EBV efficiently immortalizes the host cell and predisposes it to a number of malignancies, including Burkitt's lymphoma, nasopharyngeal carcinoma, gastric carcinoma, Hodgkin's disease and a variety of lymphomas in immunosuppressed patients [1]. In latently infected cells, replication and maintenance of the viral genome require the latent origin of replication, *oriP* and the EBNA1 protein. O*riP* is comprised of two functional elements, the dyad symmetry (DS) and the family of repeats (FR), which contain four and twenty copies of an 18 bp palindromic EBNA1 binding site respectively [2],[3]. Replication of *oriP*-containing plasmids requires EBNA1 binding to the DS [4]. EBNA1 binding to the FR is required for the mitotic segregation of the *oriP*-containing plasmids and transactivation of several latency genes [5],[6].

EBNA1 binds DNA through residues 459–607, which form the DNA binding and dimerization domain (EBNA1-DBD) [7]–[9]. High resolution structures of the EBNA1-DBD, alone and in complex with its DNA binding site, have revealed details of the interaction of EBNA1 with DNA [10]–[12]. EBNA1-DBD comprises two subdomains: residues 504–604, referred to as the core-domain, and residues 461–503, referred to as the flanking domain. The core domain is a β-barrel structure that forms the dimerization interface and makes transient sequence-specific contacts with the DNA through an α-helix [10],[13]. The flanking domain consists of an α-helix (residues 477–489) oriented perpendicular to the axis of the DNA, which contacts the major groove through Lys 477, and an extended chain (amino acids 461–469) that runs along the base of the minor groove of the DNA, making sequence-specific contacts through Lys-461, Gly-463 and Arg-469 [11].

In addition to binding specific DNA sequences, EBNA1 is also known to interact with several host-cell proteins, which in some cases have been shown to mediate EBNA1 functions at *oriP* [14]–[18]. EBNA1 can also affect cellular processes through sequestration of cellular proteins, as best exemplified by the EBNA1 interaction with the ubiquitin specific protease USP7, also referred to as Herpesvirus Associated Ubiquitin Specific Protease (HAUSP). USP7 was originally identified as a binding partner of the ICP0 protein of herpes simplex virus (HSV) [19] and, since then, several cellular targets of USP7 have been identified including the p53 tumour suppressor protein [20]–[24]. In response to genotoxic stress, USP7 binds and deubiquitylates p53 thereby protecting it from proteasome-mediated degradation. In addition to cleaving polyubiquitin chains, USP7 has been reported to reverse monoubiquitylation in some proteins (eg. p53 and FOXO4), thereby affecting their subcellular localization [25],[26]. Similarly, the *Drosophila* homologue of USP7 was found to contribute to epigenetic silencing by reversing monoubiquitylation of histone H2B, and this activity required USP7 to be in complex with guanosine 5′ monophosphate synthetase (GMPS) [27].

Our studies on the EBNA1-USP7 interaction have shown that EBNA1 binds the N-terminal domain of USP7 (USP7-NTD), which is distinct from the catalytic domain, and is the the same domain that is bound by p53 [28]. EBNA1 and p53 bind the same pocket in this domain but EBNA1 does so with an affinity that is approximately 10-fold higher than that of p53 [28],[29]. As a result, EBNA1 interferes with the binding and stabilization of p53 by USP7 and with p53-mediated apoptosis in response to DNA damage [29],[30]. In addition, we recently found that EBNA1 disrupts promyelocytic leukemia (PML) nuclear bodies (also called ND10s) in nasopharyngeal carcinoma cells by inducing the degradation of the PML proteins [30]. This activity required USP7 and the EBNA1-USP7 interaction, indicating that this interaction can modulate cellular events in addition to p53 levels.

EBNA1 deletion analysis showed that the USP7 binding sequence in EBNA1 was just N-terminal to the flanking DNA binding domain and subsequent peptide binding assays identified EBNA1 residues 436–450 as sufficient for this interaction [28],[29]. A crystal structure of an EBNA1 peptide bound to the USP7-NTD revealed multiple interactions of EBNA1 residues 442–448 with amino acids in a shallow groove of the TRAF domain formed by the USP7-NTD [29]. In particular interactions mediated by Ser447 in EBNA1 were shown to be critical for USP7 binding. Given the large size of USP7 (135 kDa) and the proximity of its binding site to the EBNA1-DBD residues that are inserted in the DNA minor groove (amino acids 461–469), we wondered whether the USP7 interaction interfered with EBNA1 binding to DNA. Here we report that, contrary to our expectations, USP7 had a large stimulatory effect on the DNA-binding activity of EBNA1 *in vitro* and can form a ternary complex with DNA-bound EBNA1. Furthermore, USP7 was found to bind GMPS, forming a complex active in histone H2B deubiquitylation, and this complex was recruited to *oriP* in EBV-infected cells resulting in decreased H2B ubiquitylation.

## Results

### Effect of USP7 on DNA binding by EBNA1 *in vitro*

**Figure 1:**
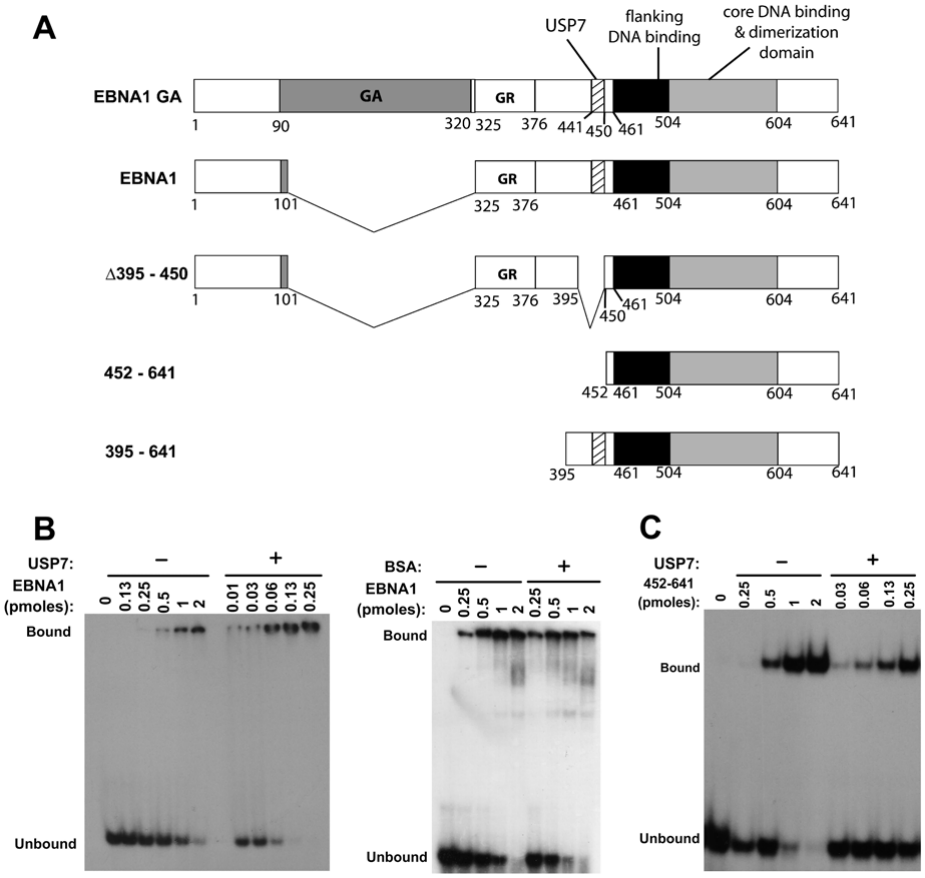
EBNA1 binding to DNA is stimulated by USP7. A. Schematic representation of EBNA1 and the EBNA1 mutants used in this study. Shown are the Gly-Ala repeat (GA), the large Gly-Arg repeat (GR), the USP7 binding site (USP7) and the flanking and core DNA binding domains. B and C. EMSAs showing titrations of EBNA1 (B) or EBNA1_452–641_ (C) with a fixed amount of DNA recognition site in the presence or absence of 10 pmols of USP7 or in the presence or absence of 10 pmols BSA as a negative control (B, right panel).

We initially assessed the effect of USP7 on the DNA binding activity of EBNA1 using electrophoretic mobility shift assays (EMSAs) with a version of EBNA1 that has a shortened Gly-Ala repeat but has wildtype activity for all known EBNA1 functions (referred to as EBNA1; Figure 1A). Purified EBNA1 was incubated with radiolabelled DNA containing a single EBNA1 recognition site (site 1 from the DS element) in presence and absence of excess purified full length USP7. We consistently observed that USP7 stimulated the DNA binding activity of EBNA1 as shown in the representative experiment in Figure 1B (left panel), while no obvious effects on EBNA1-DNA interactions were seen with nonspecific proteins such as BSA (Figure 1B, right panel). Results from multiple experiments showed a 20-fold increase in the DNA binding affinity of EBNA1 in the presence of USP7, resulting in a shift in the dissociation constant (K_d_) from 85±7nM for EBNA1 alone to 4.3±0.4 nM for EBNA1 in presence of USP7. This increase in DNA binding affinity was largely dependant on the ability of EBNA1 to bind USP7, as the DNA binding ability of a truncation mutant of EBNA1 (EBNA1_452–641_) containing the DNA-binding and dimerization region but lacking the USP7 binding site was much less affected by USP7 (on average showing a 4-fold increase in DNA binding in the presence of USP7; Figure 1C).

**Figure 2:**
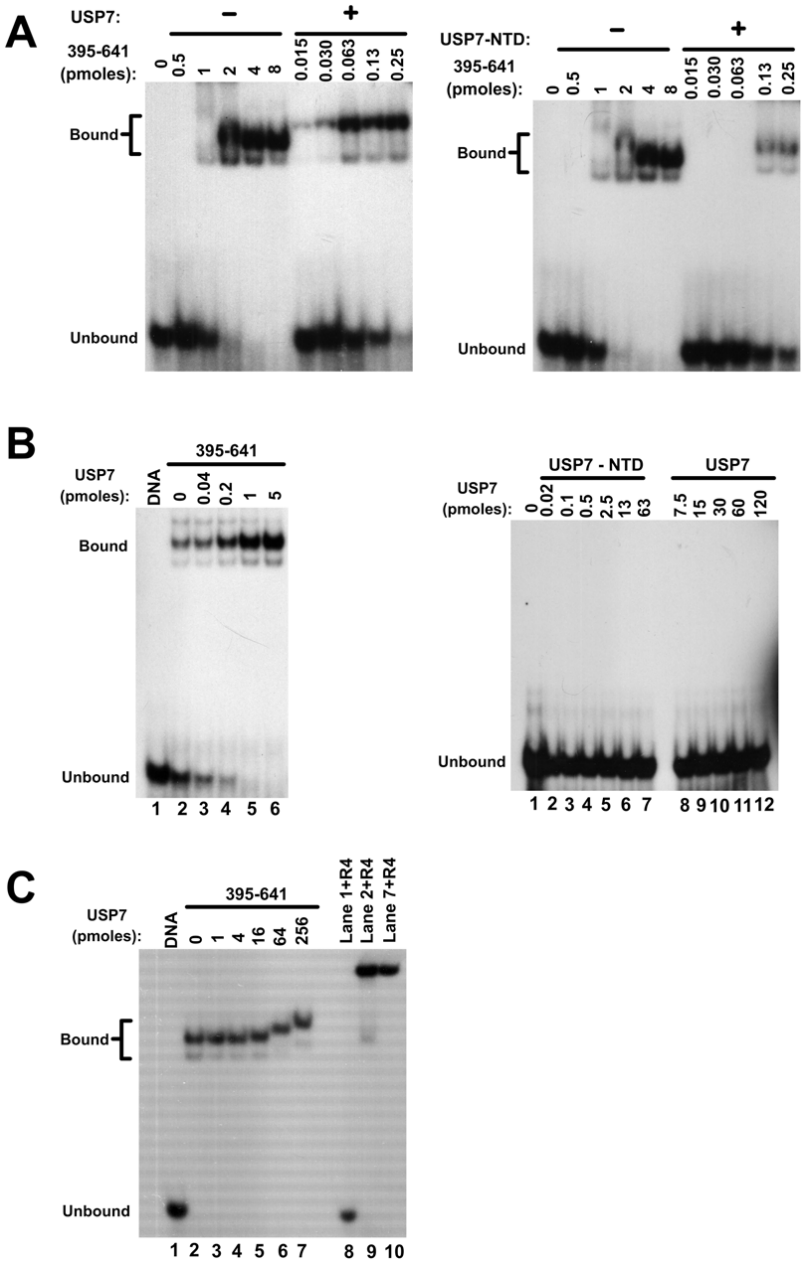
Analyses of the USP7 effect on DNA interactions of EBNA_395–641_. A. EMSAs showing titrations of EBNA_395–641_ with a fixed amount of DNA recognition site in the presence or absence of 10 pmols of USP7 (left panel) or USP7-NTD (right panel). B. EMSAs performed with a fixed amount of EBNA_395–641_ and DNA and the indicated amounts of USP7 (left panel). Titrations of USP7 and the USP7-NTD with DNA in the absence of EBNA1 are shown in the right panel. C. Complexes of EBNA_395–641_ and DNA were preformed then incubated with the indicated increasing amounts of USP7. Complexes formed as in lanes 1,2 and 7 were then incubated with anti-EBNA1 antibody (R4) prior to polyacrylamide gel electrophoresis.

EBNA1 dimers bound to DNA are known to interact with each other resulting in the crosslinking of multiple DNA fragments through large EBNA1 complexes (referred to as looping or linking interactions) [31]–[33]. These complexes are retained in the wells of the gel in EMSAs as shown in Figure 1B, precluding analysis of the effect of USP7 on the migration of the DNA complexes. The linking interactions of EBNA1 are mediated largely by amino acids 325–376 and to a lesser degree by EBNA1 N-terminal residues [32],[34]. To further evaluate the effect of USP7 on the DNA binding ability of EBNA1 without the confounding effects of DNA linking, we repeated the EMSAs with the EBNA1 truncation mutant 395–641 (Figure 1A), which contains the USP7 binding site and the DNA-binding region but lacks sequences that cause DNA linking. When the DNA binding affinity of EBNA1_395–641_ was measured in the presence and absence of excess USP7, USP7 was consistently found to stimulate DNA binding by EBNA1_395–641_ (Figure 2A, left panel), resulting in a 50-fold decrease in the calculated K_d_ from 233±76 nM for EBNA1_395–641_ alone to 4±1.8 nM for EBNA1_395–641_ in presence of USP7. This experiment also showed that the bound DNA migrated more slowly in the presence of EBNA1_395–641_ and USP7 than with EBNA1_395–641_ alone, suggesting that USP7 formed a ternary complex with EBNA1_395–641_ and DNA.

Since EBNA1 is known to bind to the N-terminal TRAF domain of USP7 (USP7-NTD) [28],[29], we examined whether this domain was sufficient to stimulate EBNA1_395–641_ binding to DNA. When EBNA1_395–641_ titrations were performed in the presence of excess USP7-NTD, the DNA binding activity was increased 8 to 16-fold in multiple experiments, (Figure 2A, right panel) indicating that the USP7-NTD was partially, but not completely, responsible for the stimulatory effect of USP7 on EBNA1 DNA binding activity. Consistent with the USP7 result, the USP-NTD was found to decrease the migration of the EBNA1-bound DNA suggesting that it can bind the EBNA1-DNA complex.

We also examined the stimulatory effect of USP7 on DNA binding by EBNA1_395–641_ by incubating a fixed amount of EBNA1_395–641_ (sufficient to bind a small fraction of the DNA probe on its own) with increasing amounts of USP7 prior to the addition of the DNA binding site Figure 2B, left panel). EMSAs performed in this way showed that USP7 had a dose-dependent effect on the DNA binding activity of EBNA1_395–641_. The possibility that USP7 itself had some ability to bind the DNA probe was tested by titrating USP7 with the DNA in the absence of any EBNA1, but USP7 alone did not shift the DNA probe even at very high concentrations of USP7 (Figure 2B, right panel lanes 8–12). Similarly, the USP7-NTD on its own did not bind the DNA-probe (Figure 2B, right panel lanes 1–7).

The experiments in Figure 2A indicated that USP7 can bind the EBNA1-DNA complex resulting in a supershift while the titration performed with lesser amounts of USP7 in Figure 2B did not show a supershift. To investigate this discrepancy, we preformed EBNA1-DNA complexes (using EBNA1_395–641_ as above) then added increasing amount of USP7 (Figure 2C). EMSAs confirmed that USP7 was able to supershift the EBNA1_395–641_-DNA complex but only at higher concentrations of USP7 (compare lanes 6 and 7 to lanes 2–5). To confirm that the supershifted band contained EBNA1, complexes formed as in lanes 2 and 7 were incubated with an EBNA1-specfic antibody prior to electrophoresis. In both cases the antibody supershifted the bands to the gel wells, whereas no effect of the antibody was seen on the migration of the DNA probe in the absence of EBNA1 (Figure 2C, lanes 8–10). The results indicate that USP7 can form a ternary complex with DNA-bound EBNA1 under some conditions.

### Effects of USP7 silencing on EBNA1-DNA interactions *in vivo*

**Figure 3:**
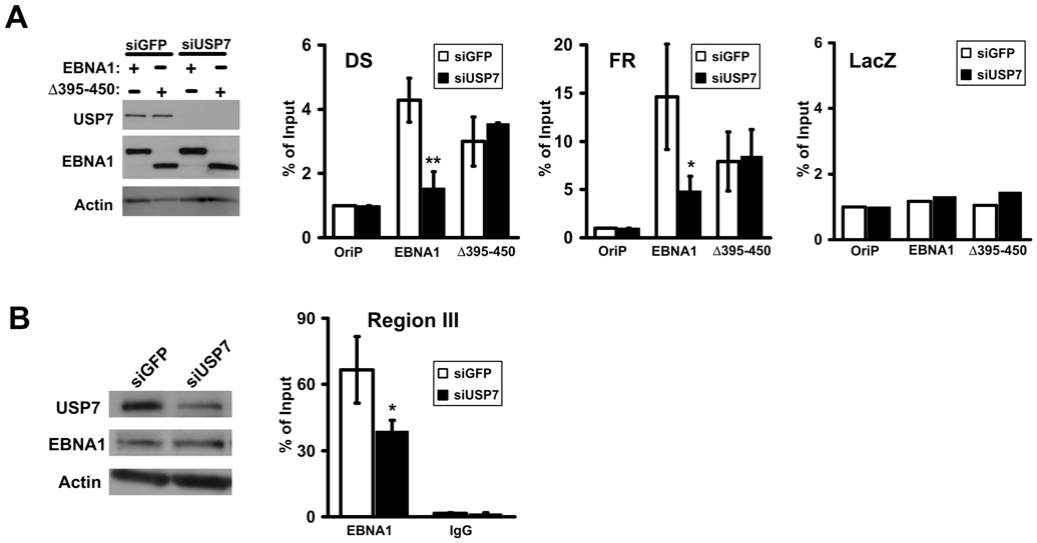
Effects of USP7 silencing on EBNA1-DNA interactions in vivo. A. CNE2Z cells were treated with siRNA against USP7 or GFP then co-transfected with pLacZ and with an oriP plasmid expressing EBNA1 or Δ395–450 as indicated or empty oriP plasmid (oriP). Equal amounts of cell lysates were analysed for protein expression by Western blotting (left panel) and ChIP assays were performed with EBNA1 and nonspecific antibodies for the DS and FR elements of *oriP* and for the lacZ gene. Results are shown after normalization to nonspecific IgG and input DNA. B. D98/Raji cells were transfected with siRNA against USP7 or GFP then ChIP assays were performed with EBNA1 antibodies and nonspecific antibodies (IgG) and a primer set near region III. Changes with P values less than 0.01 (**) and less than 0.05 (*) relative to siGFP samples are indicated.

During initial EBV infection, EBNA1 assembles on its recognition sites in *oriP* and remains stably bound to these sites in all types of latently infected cell lines. Therefore it was not possible to determine the effects of USP7 on EBNA1 assembly on *oriP* using latently infected cells. Instead, we assessed the effect of USP7 on the initial association of EBNA1 with *oriP* by treating EBV-negative nasopharyngeal carcinoma cells (CNE2Z) with siRNA against USP7 or GFP (negative control) and then transfecting these cells with an *oriP* plasmid expressing EBNA1 or an EBNA1 mutant (Δ395–450; see Figure 1A) that we previously showed was specifically defective in binding USP7 [14] and a plasmid lacking EBNA1 binding sites (pLacZ) as control for nonspecific DNA binding. Chromatin immunoprecipitation (ChIP) assays were then performed using EBNA1-specific antibodies to assess the degree of EBNA1 association with the the *oriP* FR and DS elements and *lacZ* (negative control) as compared to nonspecific rabbit IgG. EBNA1 was readily detected on both the DS and FR elements after siGFP treatment but the association with both elements was greatly decreased by USP7 silencing (Figure 3A, middle panels). As expected, there was little association of EBNA1 with *lacZ* and this was unaffected by USP7 silencing (right panel). Consistent with the *in vitro* results, Δ395–450 bound less efficiently to both the DS and FR elements than did wildtype EBNA1, despite being expressed at equivalent levels as EBNA1 (see Figure 3A, left panel). Moreover, unlike wildtype EBNA1, the interaction of Δ395–450 with the FR and DS elements was not affected by USP7 silencing. Therefore we conclude that USP7 can stimulate the assembly of EBNA1 on *oriP* elements *in vivo*.

In addition to binding the *oriP* elements, EBNA1 can interact in a more transient manner with a third region of the EBV genome (referred to as region III), consisting of two lower affinity EBNA1 recognition sites within the BamHI-Q fragment, and this interaction can negatively regulate the Qp promoter used for EBNA1 expression in some types of EBV latency [3],[35],[36]. Due to the transient nature of the EBNA1 interaction with region III, we asked whether USP7 might promote the EBNA1-region III interaction in latently infected cells. D98/Raji cells were used for these experiments since these EBV-infected cells are more transfectable than the Raji cells from which they were derived. D98/Raji cells were transfected with siRNA against USP7 or GFP then ChIP experiments were performed using EBNA1-specific antibody and primer sets for region III. While we did not achieve complete silencing of USP7 in these experiments (Figure 3B, left panel), its down-regulation was consistently found to decrease the association of EBNA1 with region III (Figure 3B, right panel), indicating that USP7 can also modulate EBNA1-DNA interactions in the context of an EBV infection.

### USP7 is recruited to EBV oriP

**Figure 4:**
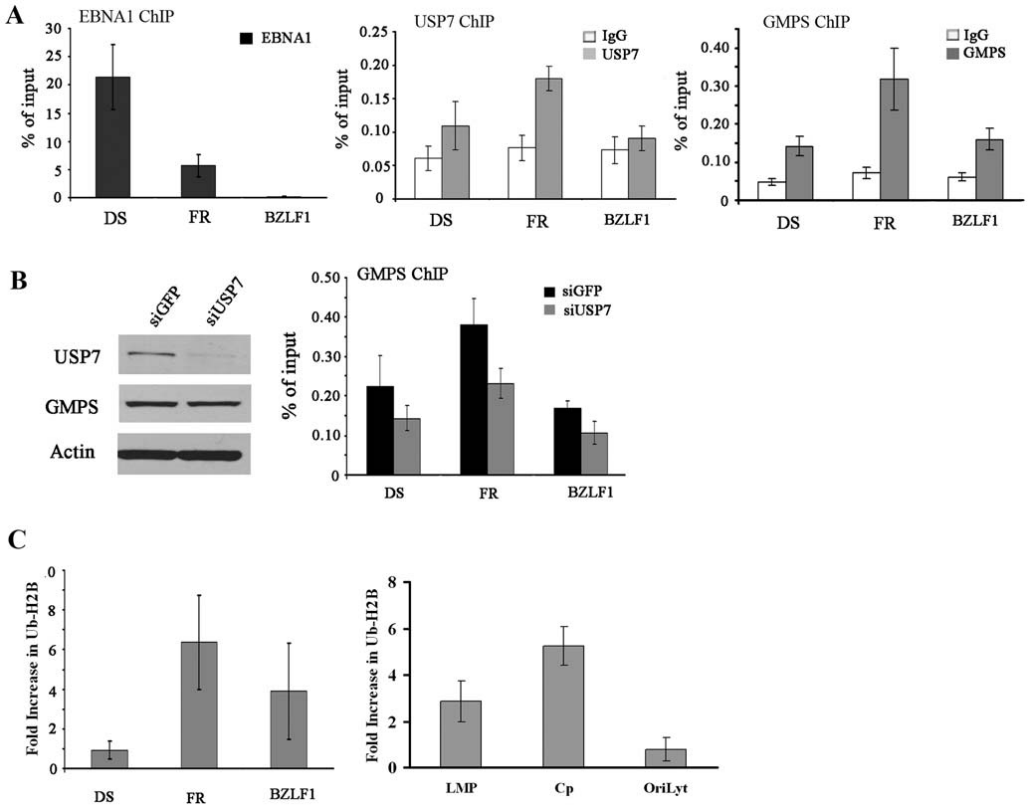
Chromatin IP assays for USP7, GMPS and Ub-H2B in EBV genomes. A. ChIP experiments were performed in Raji cells using antibodies against EBNA1 (left panel), USP7 (middle panel), GMPS (right panel) and nonspecific rabbit IgG as a negative control. Recovered DNA fragments were quantified by real-time PCR using primer sets for the *oriP* DS and FR regions or the BZLF1 promoter region. B. D98/Raji cells were treated with siRNA against USP7 or GFP (negative control), then ChIP experiments were performed as in A using antibodies against GMPS (right panel). Down-regulation of USP7 by siUSP7 treatment was confirmed by Western blotting, while GMPS levels were unaffected by this treatment (left panel). C. D98/Raji cells were treated with siRNA against USP7 or GFP and ChIP assays were performed using antibodies against histone H2B and monoubiquitylated histone H2B (Ub-H2B) and primer sets for the indicated region of the EBV genome (LMP = LMP1 promoter region). Relative ratios of Ub-H2B to total H2B were determined for each treatment and the average fold increase in Ub-H2B after siUSP7 treatment (as compared to siGFP treatment) from multiple experiments is shown.

The above i*n vitro* analyses raised the possibility that EBNA1 may recruit USP7 to *oriP* in EBV-infected cells. To test this possibility we conducted ChIP experiments in EBV-positive B-lymphocytes (Raji cells). Antibodies against EBNA1 or USP7 were used to immunoprecipitate these proteins from sheared Raji DNA and compared to non-specific rabbit IgG as a negative control. Immunoprecipitates were analyzed by quantitative real-time PCR using primers specific for the DS and FR regions in *oriP* and for the promoter region of the BZLF gene, located 40 kb away from *oriP*. EBNA1 is known to be constitutively bound to the FR and DS elements [37],[38] and, consistent with this, was readily detected on both the FR and DS DNA fragments (with better recovery of the DS element as has been previously observed;[16],[39],[40]) but was not detected on the BZLF1 fragment (Figure 4A). The USP7 antibody consistently isolated more FR DNA fragment than either the DS or BZLF1 fragments (Figure 4A). Recovery of the FR region (but not the DS region) was significantly higher than that of the BZLF1 region with a p-value of 0.0004. The results indicate that USP7 is preferentially recruited to FR and is consistent with the higher enrichment of EBNA1 at the FR.

### USP7 forms a complex with GMP synthetase that deubiquitylates histone H2B

**Figure 5:**
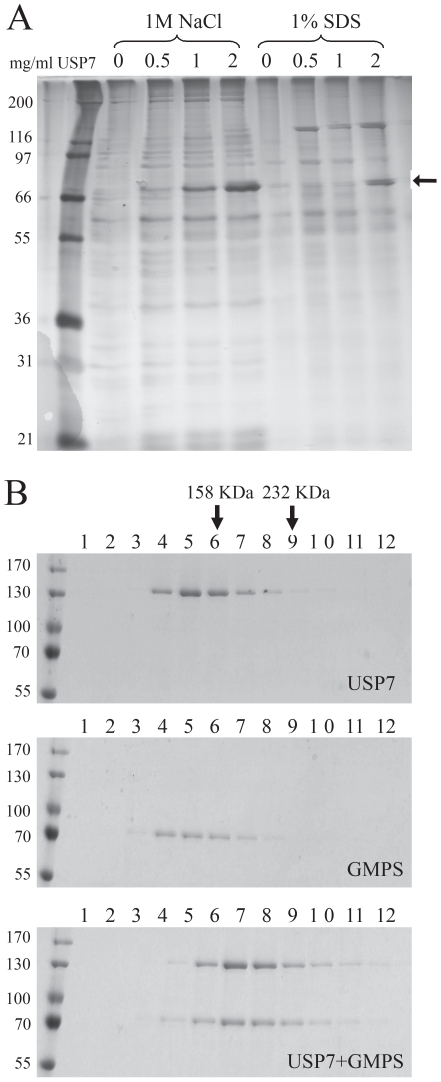
USP7 forms a complex with GMPS. A. Purified USP7 was coupled to a fixed amount of resin at the indicated concentrations to generate a series of affinity columns. A constant amount of HeLa whole cell lysate was applied to each, followed by washing then elution of the bound proteins with 1 M NaCl then with 1% SDS. A silver-stained gel is shown in which the band marked by the arrow was excised and identified as GMPS by MALDI-ToF mass spectrometry. The band at 120 kDa in the 1% SDS elution is USP7 itself. B. Purified USP7 and GMPS were analysed by glycerol gradient sedimentation individually (top and middle panels) and after mixing the two proteins (bottom panel). Equal volume fractions were collected from the top of each gradient and analysed by SDS-PAGE and colloidal Coomassie staining. The positions of 158 kDa (aldolase) and 232 kDa (catalase) molecular weight markers are indicated at the top of the gels.

USP7 is known to regulate p53 levels but this would not seem to explain why it is recruited to *oriP*. To gain insight into other potential functions of USP7, we used a proteomics approach to identify cellular protein partners of USP7. To this end, increasing amounts of purified USP7 was coupled to resin to generate a series of USP7 affinity columns and a constant amount of human cell extract was passed through each column. Proteins retained on the columns were eluted with 1 M NaCl, followed by 1% SDS, and the recovered proteins were analysed by SDS-PAGE and silver staining (Figure 5A). Only 1 band (at approximately 70 Kda) was observed to be specifically retained on the USP7 column, showing a titratable interaction with USP7 as expected for a specific protein interaction, and this was identified by MALDI-ToF mass spectrometry as GMP synthetase (GMPS).

The interaction between USP7 and GMPS was further examined by glycerol gradient sedimentation analysis of the purified proteins. For these experiments, GMPS, like USP7, was generated using a baculovirus and extensively purified. Analysis of the individual proteins by glycerol gradient sedimentation showed that USP7 migrates close to its calculated molecular mass of 130 Kd indicating that it is monomeric (Figure 5B, top panel). This is consistent with previous analytical centrifugation analyses [28]. GMPS was found to migrate at a similar position as USP7 despite its smaller molecular mass of 77 Kda suggesting that it forms dimers (Figure 5B, middle panel), as occurs for *E.coli* GMPS [41]. When USP7 and GMPS were combined, their positions in the gradient shifted to a higher molecular weight form, confirming that the two proteins directly interact (Figure 5B, bottom panel). The size of this complex (approximately 200 Kda) suggested that it consisted of one USP7 and one GMPS molecule.

**Figure 6:**
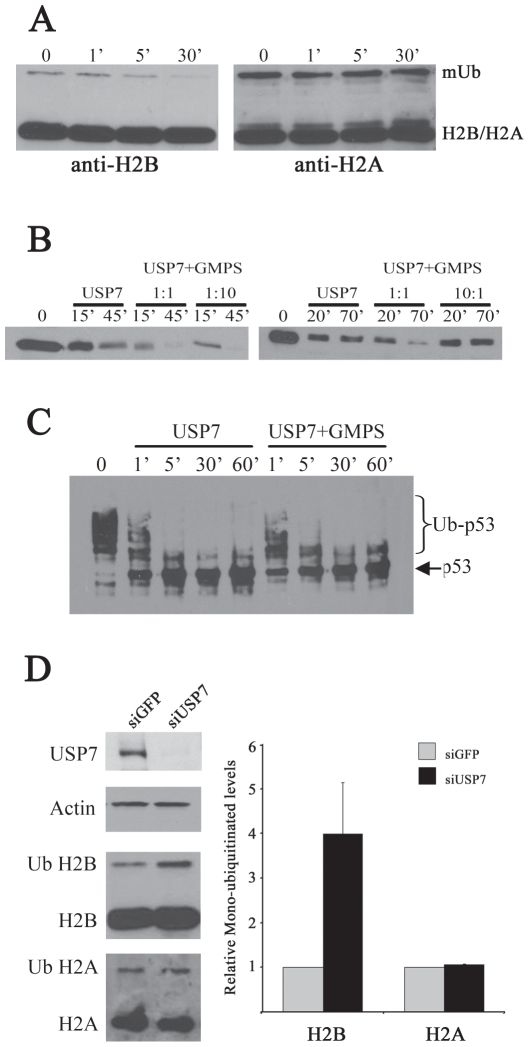
GMPS stimulates histone H2B deubiquitylation by USP7. A. Total histones isolated from HeLa cells were incubated with USP7 (1∶1000 ratio of USP7∶histones) for 0, 1, 5 or 30 minutes then analysed by Western blotting using antibodies against histones H2B (left panel) or H2A (right panel). The positions of the unmodified (H2B/H2A) and monoubiquitylated (mUb) histones are indicated. B. Total histones were incubated with USP7 as in A for the indicated number of minutes, with (USP7+GMPS) or without (USP7) GMPS, at a ratio of 1∶1, 1∶10 or 10∶1 USP7∶GMPS as indicated. Western blot analysis was then performed using anti-ubiquitin antibody and the band corresponding to monoubiquitylated H2B in part A is shown. C. Polyubiquitylated p53 was incubated with USP7 for the indicated number of minutes with (USP7+GMPS) or without (USP7) a 10-fold excess of GMPS. Samples were analysed by Western blotting using p53 antibody. The positions of unmodified (p53) and ubiquitylated p53 (Ub-p53) are indicated. D. HeLa cells were transfected with siRNA against GFP or USP7 and USP7 silencing was confirmed by Western blotting of whole cell extracts as compared to an actin loading control (top two gel panels). Total histones were prepared from the siRNA treated cells and Western blots were performed using antibodies against histones H2B or H2A (bottom two gel panels). The ratio of the monoubiquitylated to unmodified forms was determined for H2A and H2B and the results from multiple experiments are shown in the histogram, in relationship to the ratio observed with siGFP treatment (set to 1).

A previous study reported that *Drosophila* USP7 formed a complex with GMPS in *Drosophila* embryos and that this complex deubiquitylated histone H2B thereby contributing to polycomb-mediated silencing [27]. This prompted us to investigate whether the human USP7-GMPS complex also functioned to deubiquitylate histone H2B. To this end, we purified total histones from HeLa cells by the acid extraction method and incubated them with purified USP7 (at a MW ratio of USP7∶histones of 1∶1000) for various times prior to Western blot analysis. Histone H2B and its monoubiquitylated form (Ub-H2B) were initially detected using an antibody specific to histone H2B, and USP7 was found to have some ability to deubiquitylate H2B on its own (Figure 6A, left panel). Histone H2A and its monoubiquitylated form were detected in the same assay with antibody specific to H2A, however, in contrast to the H2B results, USP7 was not observed to deubiquitylate H2A (Figure 6A, right panel).

To determine if GMPS affected the ability of USP7 to deubiquitylate H2B, we repeated the experiments including different amounts of GMPS (Figure 6B). The Ub-H2B was more readily detected using an anti-ubiquitin antibody, providing a more robust signal to follow and this band is shown in Figure 6B. We found that the addition of GMPS at amounts stoichiometric to USP7 increased the cleavage of Ub-H2B by USP7 at each time point examined (compare “1∶1” samples to “USP7” samples within each panel). Increasing the amount of GMPS 10-fold had no further stimulatory effect (compare “1∶10” samples to “1∶1” samples in the left panel), while decreasing the amount of GMPS 10-fold abrogated the stimulatory effect (compare “10∶1” samples to “1∶1” samples in the right panel). These results are consistent with GMPS stimulating deubiquitylation of H2B by USP7 by forming a stoichiometric complex with USP7 and are inconsistent with GMPS acting catalytically. We also asked whether the stimulatory activity of GMPS was specific to H2B deubiquitylation or also occurred for other USP7 targets. To this end, we incubated USP7, with or without equal amounts of GMPS, with p53 that had been polyubiquitylated *in vitro* and we followed the p53 forms by Western blotting with a p53 antibody (Figure 6C). In this case, we saw no obvious difference in the kinetics of cleavage of the ubiquitylated forms by USP7 with or without GMPS, indicating that GMPS does not affect all USP7 targets equally and rather has specificity for Ub-H2B.

To assess whether USP7 regulates histones in human cells, we down-regulated USP7 in HeLa cells with siRNA treatment then prepared total histones as for the *in vitro* assays. The ratio of monoubiquitylated to nonmodified forms of H2A and H2B were then determined by Western blotting using antibodies against H2A and H2B. An example of the results obtained is shown in the gel images in Figure 6D as compared to results with the same cells treated with siGFP as a negative control. We consistently observed an increase in the ratio of Ub-H2B to total H2B after USP7 silencing, as compared to GFP silencing (negative control), but we did not see a reproducible effect on the H2A monubiquitylated form. Results from three independent experiments are shown in histogram in Figure 6D. Therefore the *in vivo* studies support the conclusions of the *in vitro* results, that USP7 can regulate H2B monoubiquitylation.

### Formation of a DNA-EBNA1-USP7-GMPS quaternary complex

**Figure 7:**
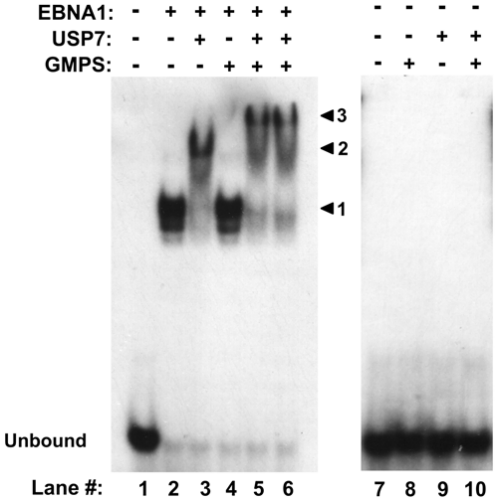
GMPS can form a quaternary complex with USP7, EBNA1 and DNA. The indicated combinations of EBNA1_395–641_ , USP7 and GMPS were preincubated then combined with the DNA containing the EBNA1 recognition site and EMSAs were performed as in Figure 2C. Excess amounts of USP7 alone or USP7 and GMPS were used relative to EBNA1_395–641_. In lane 6, the USP7-EBNA complex was preformed prior to the addition of GMPS then DNA. The positions of complexes formed by EBNA1 alone, EBNA1+USP7 and EBNA1+USP7+GMPS are indicated by arrowheads 1, 2 and 3 respectively. DNA incubated with the same amounts of GMPS, USP7 or GMPS+USP7 but in the absence of EBNA1 are also shown (lanes 8–10).

We next investigated the relevance of the USP7-GMPS interaction for EBNA1, in particular whether GMPS could form part of the USP7-EBNA1-DNA complex. We examined this in two ways: First, we tested possible interactions between DNA-bound EBNA1_395–641_ with GMPS with and without USP7 by EMSAs (Figure 7). The binding of EBNA1_395–641_ to the DNA probe was assessed on its own or after incubation of the same amount of EBNA1 with USP7 or GMPS and the migration of the DNA complexes was assessed. As observed above, USP7 shifted the EBNA1-DNA complex to a slower migrating form indicative of a ternary complex (Figure 7, compare lanes 2 and 3). On the other hand, the same amount of GMPS did not alter the mobility of the EBNA1-DNA complexes (Figure 7, compare lanes 2 and 4). This was expected since there is no evidence of a direct interaction between EBNA1 and GMPS. However, when USP7, GMPS and EBNA1 were combined (the same amounts as when tested individually), and then added to the DNA, these complexes shifted to a position higher than that of the USP7-EBNA-DNA ternary complex as shown in lanes 5 and 6 of Figure 7 (compare to lane 3). However neither GMPS, USP7 nor GMPS+USP7 interacted with the DNA in the absence of EBNA1 (Figure 7, lanes 8–10). The results suggest that USP7 mediates an interaction between GMPS and the EBNA1-DNA complex resulting in the formation of a quaternary complex.

We also examined the possible association between USP7-GMPS complexes and EBNA1 *in vivo*, by determining if GMPS localized with EBNA1 and USP7 on EBV chromatin. ChIP experiments performed on Raji cells, showed that, like USP7, GMPS was preferentially detected at the FR element of *oriP* over the DS element or the BZLF1 region (Figure 4A, right panel). This is consistent with the recruitment of the USP7-GMPS complex to the FR through EBNA1.

We next investigated whether recruitment of GMPS to the FR was dependent on USP7, as suggested by the EMSA experiments. These experiments required down-regulation of USP7 by siRNA treatment and could not be performed in Raji cells due to their low transfection efficiency. Instead, the more readily transfectable D98/Raji fusion cells were used, which retain the EBV genomes from Raji cells [42]. USP7 was confirmed to be down-regulated in these cells following treatment with siRNA against USP7 but not siRNA against GFP (negative control), while GMPS levels were not affected (Figure 4B, left panel). ChIP analysis of GMPS from these cells showed that, as in Raji cells, GMPS was preferentially localized to the FR region, and that down-regulation of USP7 resulted in decreased levels of GMPS at the FR (P value 0.01 relative to FR-siGFP samples; Figure 4B, right panel).

If the USP7-GMPS complex functions to deubiquitylate histone H2B, then the loss of this complex from the FR would be expected to increase the level of Ub-H2B in this region. We investigated this possibility by performing ChIP experiments with and without USP7 silencing, using an antibody that recognizes only the ubiquitylated form of H2B [43]. To control for possible differences in the number of histones at each region we performed the same experiment with antibody against total histone H2B and expressed the Ub-H2B as a ratio of this value. In Figure 4C (left panel) the change in the fraction of Ub-H2B after USP7 silencing is shown from multiple experiments (in relation to siGFP treatment). While we saw considerable variability on the level of Ub-H2B at the BZLF1 region, we consistently observed that USP7 silencing resulted in increased levels of Ub-H2B at the FR and had little effect on Ub-H2B levels at the DS. The results support the model that USP7 is needed for recruitment of GMPS to the FR and subsequent deubiquitylation of histone H2B.

Since EBNA1 binding to the FR is known to activate transcription from the LMP1 and Cp promoters [44],[45], we examined the possibility that the recruitment of the USP7-GMPS complex to the FR might also affect H2B ubiquitylation at these promoters. To this end, ChIP was performed on D98/Raji cells before and after silencing USP7, using antibodies against Ub-H2B and total H2B. The recovery of the LMP1 and Cp promoter regions was quantified for each treatment and the change in the fraction of Ub-H2B after USP7 silencing was determined. Silencing of USP7 consistently resulted in increased Ub-H2B at both the LMP1 and Cp promoters, with the strongest effect on the Cp promoter, whereas H2B ubiquitylation at the oriLyt region of EBV (negative control) was not affected by USP7 silencing (Figure 4C, right panel). The results suggest that the USP7-GMPS complex not only affects H2B ubiquitylation at the FR but also at promoters controlled by the FR.

### USP7 contributes to transcriptional activation by EBNA1

**Figure 8:**
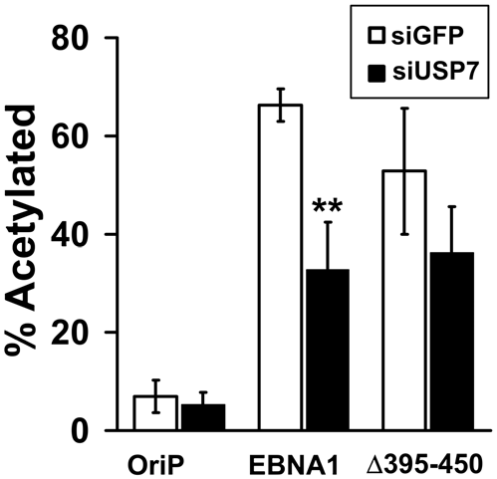
Effect of USP7 silencing on EBNA1-mediated transcriptional activation. CNE2Z cells were treated with siRNA against USP7 or GFP then were co-transfected with an FR-CAT reporter plasmid and an oriP plasmid expressing EBNA1, Δ395–450 or no EBNA1 (oriP). CAT assays were then performed on equal amounts of cell lysates and the percent of acetylated substrate was determined as a measure of transcriptional activation. Changes with P values less than 0.01 (**) and less than 0.05 (*) relative to siGFP samples are indicated.

The above observations suggest that EBNA1-mediated recruitment of the GMPS-USP7 complex to the FR may contribute to transcriptional activation by this element through alteration of Ub-H2B at the FR and/or promoters under FR control. To test this possibility, we treated EBV-negative CNE2Z cells with siRNA against USP7 or GFP then co-transfected them with a reporter plasmid in which expression of chloramphenical acetyl transferase (CAT) is under FR control and with a plasmid expressing either EBNA1, the EBNA1 Δ395–450 mutant that is unable to bind USP7 or no EBNA1 (oriP plasmid). CAT assays were then performed on each sample to assess degree of transcriptional activation (Figure 8). As expected strong transcriptional activation was seen after siGFP treatment in the presence of EBNA1 but not in its absence and, as previously reported [14], Δ395–450 had slightly reduced transcriptional activity. USP7 silencing caused a significant decrease in transcriptional activation by EBNA1 (P value 0.004) but did not significantly affect transactivation by Δ395–450. These results support the model that recruitment of the USP7-GMPS complex by EBNA1 contributes to EBNA1-mediated transcriptional activation.

## Discussion

EBNA1 forms a stable complex with host cell USP7 and this interaction can promote cell survival, at least in part through interfering with p53 stabilization by USP7 and through disrupting PML nuclear bodies [14], [28]–[30]. Here we provide the first evidence that the EBNA1-USP7 interaction also contributes to EBNA1 functions at EBV *oriP*. This study stemmed from the unexpected observation that USP7 greatly stimulated the DNA binding activity of EBNA1 *in vitro* and could form a ternary complex with DNA-bound EBNA1. EBNA1 appears to be constitutively bound to *oriP* elements in latent EBV infections in proliferating cells [37],[38] and, in these cases, the functional relevance of these observations for *oriP*-related functions most likely lies in the ability of USP7 to form a ternary complex with DNA-bound EBNA1, as verified at the FR element in EBV-infected cells. In keeping with this hypothesis, we found that USP7 within this complex can mediate an interaction with GMPS which promotes deubiquitylation of histone H2B and that USP7 contributes to EBNA1-mediated transcriptional activation. However we have also shown that USP7 can stimulate the assembly of EBNA1 on *oriP* elements in transfected plasmids suggesting that USP7 might play a role in the initial association of EBNA1 with these elements upon initial EBV infection, and/or during the switch from the EBV latency form in nonproliferating cells, in which EBNA1 is not expressed (referred to as the latency program [46]), to latency forms in proliferating cells in which EBNA1 is expressed and bound to *oriP*. In addition, we have shown that USP7 can stimulate EBNA1 binding to region III in the EBV genome which, under some circumstances, negatively regulates EBNA1 expression [35],[36], raising the possibility of a role for USP7 in EBNA1 autoregulation from the Qp promoter.

We have previously shown that EBNA1 residues 441–450 bind to the USP7-NTD [28],[29]. The ternary complex formed between USP7 and DNA-bound EBNA1 also appears to require the interaction of the USP7-NTD with the EBNA1 441–450 region for the following two reasons. First, the USP7-NTD was sufficient to supershift the EBNA1-DNA complex. Second, USP7 did not supershift the complex formed by DNA and EBNA1_452–641_, which lacks the USP7 binding site but retains full DNA binding activity. However, it is curious that we observed partial but not complete stimulation of EBNA1 DNA binding by the USP7-NTD. We had previously assessed the ability of all USP7 stable domains to bind EBNA1 by examining the retention of partially proteolysed USP7 on an EBNA1 affinity column and only the USP7-NTD was found to bind EBNA1 [28]. However, this does not eliminate the possibility that other regions of USP7 might have weak affinities for EBNA1. Our *in vitro* data are consistent with a model in which the USP7-NTD binds EBNA1 residues 441–450 to bring USP7 to EBNA1, enabling subsequent weaker or less specific interactions of other regions of USP7 with the EBNA1 DNA binding or C-terminal regions (452–641). This might explain why the DNA binding activity of EBNA1_452–641_ was weakly stimulated by USP7. Another possible interpretation of the *in vitro* data is that the interaction of the USP7-NTD with EBNA1 is stabilized by the rest of USP7 due to effects on the structure of the USP7-NTD. However we do not think this is likely because the USP7-NTD is a TRAF domain that is stably folded in the absence of the rest of USP7 [29],[47].

While stoichiometric amounts of USP7 were sufficient to stimulate the DNA binding activity of EBNA1, only at higher USP7 concentrations was USP7 observed to be stably associated with the EBNA1-DNA complex *in vitro*. This indicates that the affinity of USP7 for free EBNA1 is higher than for DNA-bound EBNA1 and that a higher effective concentration of EBNA1 or USP7 may be necessary to drive the interaction of these proteins on DNA. This conclusion is also supported by the observation that USP7 is preferentially associated with EBNA1 on the FR element over EBNA1 on the DS element of *oriP*. The FR element is bound by 20 EBNA1 dimers as compared to 4 EBNA1 dimers at the DS element and, in both cases, the dimers within the element interact with each other to form a larger EBNA1 complex [31],[32]. As a result the effective concentration of EBNA1 at the FR is higher than at the DS and this may drive recruitment of USP7.

An increasing number of human cellular protein binding targets of USP7 have been identified including p53, Mdm2, FOXO, March 7 and PTEN, all of which can be deubiquitylated by USP7 [20]–[24],[26]. Our proteomic profiling of USP7 protein interactions identified GMPS as another USP7 binding partner. We expect that other USP7 binding partners were not identified by this method due to their low abundance or transient nature of the interaction in response to particular stimuli (such as occurs with the USP7-p53 and USP7-FOXO interactions). The interaction of USP7 with GMPS is unique in that it appears to affect the activity of USP7 for specific substrates, as opposed to being a substrate itself. This is supported by the fact that GMPS levels are not altered when USP7 is silenced (as shown in Figure 4B).

The finding that human USP7 forms a stable complex with GMPS fits well with the observations of van der Knaap et al [27], where *Drosophila* USP7 was found to co-purify with GMPS. Our glycerol gradient sedimentation analyses indicated that human USP7 and GMPS form a 1∶1 complex and *in vitro* assays show that GMPS stimulates the ability of USP7 to deubiquitylate H2B (but not H2A), as observed for the *Drosophila* GMPS-USP7 complex. Van der Knapp et al [27] also showed that the stimulation of *Drosophila* USP7 activity by GMPS did not require the catalytic activity of GMPS. Our *in vitro* results are consistent with this conclusion because stimulation of USP7 deubiquitylation activity for H2B required stoichiometric amounts of GMPS (indicative of formation of a USP7-GMPS complex) and did not occur with substoichiometric amounts of GMPS (as would be expected for an enzymatic activity). Although our results are largely in agreement with those of van der Knaap et al [27], there are subtle differences in the findings of the two studies. First, *Drosophila* USP7 was not found to deubiquitylate H2B *in vitro* in the absence of GMPS while we found that human USP7 was able to cleave Ub-H2B *in vitr*o but that this activity was stimulated by GMPS. Second, In *Drosophila*, GMPS was found to stimulate deubiquitylation of p53 by USP7 and we have not observed this effect with human USP7. It is presently unclear whether these discrepancies are the result of the different *in vitro* reaction conditions and protein concentrations or reflect genuine differences in the *Drosophila* and human USP7.

ChIP assays consistently showed higher recruitment of USP7 and GMPS to the *oriP* FR over the DS and the BZLF1 promoter region, however some degree of interaction of USP7 and GMPS was also detected at the DS and BZLF1 regions as compared to the IgG negative control. This may indicate that these proteins are wide spread on chromatin where they could regulate multiple processes that are affected by H2B ubiquitylation [48]. H2B monoubiquitylation has been reported to be associated with increased transcription through effects on both initiation and elongation [43], [49]–[51], however in some instances H2B monoubiquitylation appears to inhibit transcription [52]–[55]. Therefore the contribution of H2B monoubiquitylation to gene expression is complicated and possible contributions to other DNA processes such as DNA replication are largely unexplored. We have observed that USP7 silencing increases H2B ubiquitylation at the FR as well as at LMP1 and Cp promoters and decreases transcriptional activation from the FR element, suggesting that H2B ubiquitylation is inhibitory to transcription controlled by the FR. This is consistent with our previous observation that the EBNA1 mutant that fails to bind USP7 has decreased transcriptional activation function [14].

The increased detection of USP7 and GMPS at the FR element and their effect on Ub-H2B levels in this region, suggests that EBNA1 can employ the USP7-GMPS complex for its own purposes, at least in part by decreasing the level of Ub-H2B. In addition to functioning in transcriptional activation, the EBNA1-bound FR element mediates the segregation of the EBV episomes in mitosis [5],[6],[16],[56], may enhance DNA replication from the DS [2],[57] and causes an impediment to replication fork progression [4],[58],[59]. It is conceivable that any of these processes could be affected by the state of H2B ubiquitylation, since EBV genomes in latent infection are known to exist as nucleosomal arrays [60]. We have previously shown that EBNA1Δ395–450 that does not bind USP7 has increased DNA replication activity [14], suggesting that H2B monobiquitylation could promote DNA replication but other interpretations are also possible.

Histone modifications at *oriP* are just beginning to be examined and so far these studies have been focused on histone H3 acetylation and methylation of the *oriP* DS region. Acetylated histone H3 is generally enriched at the DS but a decrease was observed at late G1 that appears to account for the delayed replication of EBV genomes [61],[62]. Histone H3 dimethyl K4 was also enriched at the DS region while H3 methyl K9 was decreased at this region [61],[63]. Our findings indicate that monoubiquitylation of H2B is another histone modification that is modulated at *oriP* and that this modification is affected by EBNA1. We had previously shown that EBNA1 binding to USP7 serves to alter cellular processes in order to facilitate cell survival [29],[30]. We now present evidence that the USP7 interaction is not limited to soluble EBNA1 but also occurs with EBNA1 bound to EBV episomes where it could regulate the plasmid maintenance and transcriptional functions of EBNA1 in EBV latent infection.

## Materials and Methods

### EBNA1 purification

EBNA1_395–641_ was expressed fused to a hexahistidine tag at the N-terminus in *Escherichia coli* from plasmid pET15b. This construct was generated by PCR amplification of EBNA1 sequences encoding amino acids 395–641 from pc3*oriP*EBNA1 and ligation between the Nde1 and BamH1 sites of pET15b. BL21 pLysS cells containing pET15b- EBNA1_395–641_ were grown to OD_600nm_ of 0.5 then induced for 3 hrs at 37°C by the addition of IPTG (0.1 mM final concentration). Cells were lysed in 50 mM NaH_2_PO_4_ pH 8.0, 300 mM NaCl, 10 mM imidazole, 20 mM β-mercaptoethanol, 0.5 mM PMSF, 1 mM benzamidine and EBNA1_395–641_ was purified on Ni-NTA Agarose resin (Qiagen) then dialyzed against 50 mM Tris pH 7.5, 300 mM NaCl, 20 mM β-mercaptoethanol, 1 mM PMSF. EBNA1_452–641_ was purified from *E.coli* as previously described [64]. EBNA1 (lacking most of the Gly-Ala repeat) was purified from insect cells as described previously [14].

### Purification of USP7 and GMPS

Full length USP7 and USP7-NTD containing amino acids 56–205 were purified as according to Holowaty et al [14]. GMPS was expressed in insect cells from a baculovirus. The GMPS baculovirus was constructed by PCR amplification of full-length GMPS cDNA in pOTB7 (ATCC number 7515509) using the primers: GCAGGATCCCATATGGCTCTGTGCAACGGAGAC (N-terminus) and GCACTCGAGTTACTCCCACTCAGTAGTTCC (C-terminus). The amplification product was digested with BamHI and XhoI and cloned between the same sites of pFastBac HT B (invitrogen). Bacmids were obtained by transformation of competent DH10Bac *E. coli* (invitrogen) with GMPS pFastBac HT B, then Spodoptera frugiperda (SF9) insect cells were transfected with the bacmids to generate the baculovirus according to manufacturer's specifications. Culture media containing the baculovirus was harvested 5 days post-transfection and amplified twice. To generate GMPS for purification, ten 15 cm plates of High Five cells at 80% confluency were infected with the GMPS baculovirus. Cells were harvested 50 hrs post-infection, washed with PBS and lysed in 10 mls of 20 mM Tris-HCl pH 8, 0.5 mM DTT, 0.5 mM EDTA, 10% glycerol and complete protease inhibitor cocktail (Roche). The lysate was sonicated, incubated 30 min on ice, then clarified by centrifugation at 64,000×g for 15 min at 4°C. The clarified lysate was incubated with 250 µl of a nickel resin (Sigma) for 1 h (with rotation) then transfered to a column. The resin was washed 3 times with 4 column volumes of column buffer (50 mM NaH_2_PO_4_, 300 mM NaCl and 10 mM imidazole) and the His-tagged GMPS was eluted from the column with column buffer containing 250 mM imidazole. EDTA and DTT were added to the elutions to a final concentration of 10 mM and the eluted protein was dialyzed overnight against 50 mM HEPES pH 7.9, 50 mM NaCl, 10% glycerol, 0.1 mM EDTA and 0.1 mM DTT then stored in aliquots at −80°C.

### Electrophoretic mobility shift assays (EMSAs)

DNA probes for EBNA1 EMSAs were generated by end-labeling a 20-mer oligonucleotide corresponding to site 1 of the DS element (5′-CGGGAAGCATATGCTACCCG-3′) with γ-^32^P-ATP and annealing it to its complementary sequence. In assays containing EBNA1 and either USP7 or GMPS, EBNA1 was preincubated with USP7 or GMPS at room temperature (RT) for 10 minutes prior to adding the labeled DNA, except in Figure 2C, where EBNA1 was incubated with labeled DNA for 10 minutes at RT first, followed by addition of increasing amounts of USP7 and further incubation at RT for 10 minutes. In Figures 1 and 2A, 10 pmols of USP7 was used along with the indicated amounts of EBNA1. For samples containing EBNA1 and both USP7 and GMPS, USP7 and GMPS were preincubated together at 4°C for 5 minutes before the addition of EBNA1 and further incubation at RT for 10 minutes. The EMSAs in Figure 7 used 2 pmol EBNA1 dimer and 64 pmols of USP7 and GMPS. Protein mixtures were incubated with 10 fmoles of labeled DNA at RT for 10 minutes in the presence of 1 µg salmon sperm DNA in 20 µl binding buffer (20 mM Tris pH 7.5, 200 mM NaCl). 4 µl of 6× DNA Loading Dye (10 mM Tris-HCl pH 7.6, 0.03% bromophenol blue, 0.03% xylene cyanol FF, 60% glycerol, 60 mM EDTA; MBI Fermentas, R0611) was then added to the reactions prior to electrophoresis on a 10% polyacrylamide gel. Bands were visualized by autoradiography.

### USP7 affinity column

Purified USP7 was covalently coupled to Affi-Gel 10 (Bio-Rad) at concentrations of 0, 0.5, 1 or 2 mgs per ml of resin in 50 mM HEPES pH 7.5, 50 mM NaCl, 1 mM DTT, 5% glycerol. The resin was then blocked in ethanolamine, equilibrated in column buffer (50 mM HEPES pH 7.5, 100 mM NaCl, 1 mM DTT, 0.1 mM EDTA, 10% glycerol) and used to generate 40 µl microcolumns as previously described [14],[65]. Whole HeLa cell lysates were generated as in Holowaty et al [14] and equal amounts were applied to each microcolumn. The columns were washed in column buffer then sequentially eluted in column buffer containing 1 M NaCl then the same buffer containing 1% SDS. Column eluates were analysed by SDS-PAGE and silver staining. The band running at 70 kDa was excised and prepared for MALDI-ToF mass spectrometry analysis as previously described [14]. Recovered peptides were analysed on a Voyager DE-STR instrument (Applied Biosystems) and the protein was identified by mass fingerprinting using ProFound software.

### Glycerol gradient analysis

50 µg of purified USP7 was incubated with 25 µg of purified GMPS in a total volume of 25 µl of 50 mM HEPES pH 7.9, 50 mM NaCl, 10% glycerol, 0.1 mM EDTA, 0.1 mM DTT for 1 hour at room temperature. Control samples were also generated in which USP7 or GMPS were incubated individually. The mixtures were then diluted to 500 µl in 50 mM HEPES pH 7.9, 5% glycerol, 200 mM NaCl and 0.5 mM EDTA and loaded onto 11.5 ml 10%–20% glycerol gradients formed in the same buffer. Gradients were subjected to centrifugation in a SW41 rotor at 34,000 rpm for 18 hours at 4°C. Fractions of 500 µl were collected from the top of each gradient and 30 µl of each fraction was analyzed on an 8% SDS-polyacrylamide gel. Proteins were visualized by colloidal blue staining. Aldolase and catalase were analyzed on identical gradients as size markers.

### *In vitro* histone deubiquitylation assays

Histones for *in vitro* assays were prepared by acid extraction as described by Kao and Osley [66]. Briefly, HeLa cells at 70% confluence were lysed in 10 mM HEPES pH 7.9, 1.5 mM MgCl, 10 mM KCl, 0.5 mM DTT, 1.5 mM PMSF and 1 mM NEM, then hydrochloric acid was added to a final concentration of 0.2 M. The lysate was incubated on ice for 30 min, then subjected to centrifugation at 10,000×g for 10 min at 4°C. The supernatant fraction, containing the histones, was dialyzed against 0.1 M acetic acid, then against distilled water and store at −70°C. Prior to use, the histones were diluted to 1 mg/ml and adjusted to a final concentration of 50 mM HEPES pH 7.9, 100 mM NaCl and 1 mM DTT. 200 µg of histones were incubated at 37°C with 0.2 µg USP7, with or without 0.1 µg GMPS (1∶1 USP7∶GMPS), 0.01 µg GMPS (10∶1 USP7∶GMPS) or 10 µg GMPS (1∶10 USP7∶GMPS) as indicated in a 200 µl reaction. Samples were collected at the indicated times and mixed with SDS-PAGE loading buffer to stop the reactions. Samples were analysed by electrophoresis on 15% SDS-polyacrylamide gels and the levels of ubiquitinated H2B and H2A were visualized by Western blotting using antibodies against H2B (Upstate Biochemicals), H2A (Upstate Biochemicals) and ubiquitin (Sigma).

### *In vitro* p53 deubiquitylation assay

Ubiquitylated p53 was generated by *in vitro* reactions with Mdm2. To this end human p53 and Mdm2 were cloned into pET15b (Novagen), expressed in *E.coli* and purified by virtue of the hexahistidine tag using standard metal affinity purification procedures. P53 was ubiquitylated *in vitro* as previously described [67]. Briefly, 5 µg p53 and 5 µg Mdm2 were incubated for 90 min at 30°C with 500 ng E1 (Calbiochem), 1 µg UbE2D2 (Boston Biochem), 50 µg ubiquitin (Boston Biochem) in a 200 µl reaction mixture containing 50 mM Tris pH 7.6, 5 mM MgCl_2_, 2 mM ATP, 2 mM DTT. Ubiquitylation was confirmed by Western blot analysis of a 10 µl sample using p53 monoclonal antibody PAb1801 [68] and the remaining mixture was stored at −80°C. For deubiquitylation assays, 5 µg (10 µl), poly-ubiquitylated p53 was incubated with 0.5 µg USP7 with or without 5 µg of GMPS in an 10 µl reaction. The samples were collected at the indicated time points, mixed with SDS-PAGE loading buffer and subjected to 10% SDS-PAGE. p53 was detected by Western blotting using p53 antibody PAb1801.

### Measurement of ubiquitylated histone levels *in vivo*

HeLa cells were transfected 3 times during a seven day period with USP7 siRNA (100 pmols, 200 pmols and 200 pmols, respectively) or with negative control siRNA against GFP [30] using Lipofectamine 2000 (Invitrogen). USP7 siRNA sequence was CCCAAATTATTCCGCGGCAAA as described in Tang et al 2006 [69]. Cells were then harvested and split into two equal samples. One sample was used to verify USP7 silencing by Western blotting using rabbit serum against USP7 [30] and anti-actin antibody (Calbiochem) as a loading control. The other sample was used to isolate the histones by acid extraction as described above and to quantify the levels of ubiquitylated histones H2B and H2A by Western blotting for these histones as described above. In each case, the amount of ubiquitylated histone was determined by normalizing the intensity of this band to that of the unmodified histone band (set to 1).

### Chromatin immunoprecipitation (ChIP) assays performed on EBV genomes

ChIP assays were performed for GMPS and USP7 in the EBV-positive, Raji Burkitt's lymphoma cells as previously described [16] using anti-USP7 rabbit antibody (Bethyl Laboratories.Inc) or rabbit antiserum raised against full length recombinant GMPS purified from insect cells. Rabbit IgG (Santa Cruz) and anti-EBNA1 R4 rabbit antibody [14] were also used as negative and positive controls, respectively. Quantitative real-time PCR was performed with a Platinum SYBR Green qPCR superMix-UDG (Invitrogen) in a Rotorgene qPCR System (Corbett Research), using 1/50^th^ of the ChIP samples or 1/2500^th^ of DNA samples prior to immunoprecipitation (input) and the previously described primer sets for the DS and FR elements and the BZLF1 promoter region [16]. Values obtained for ChIP samples were normalized to input samples with the same primer sets. For ChIP assays involving USP7 depletion, D98/Raji cells [42] were subjected to three rounds of transfection (every 24 hours) with siRNA against USP7 or with siRNA against GFP as described above. Samples were prepared as for the ChIP experiments in Raji cells except that antibodies against EBNA1, histone H2B (Upstate Biochemicals) and mono-ubiquitylated histone H2B (MediMabs Inc, Montreal) were used. Primer sets used to assess recovery of the LMP1 promoter region were CAATCAGAAGGGGGAGTGCG and ACAGCCTTGCCTCACCTGAAC, of Cp promoter region were AACCTTGTTGGCGGGAGAAG and GGCGAATTAACTGAGCTTGCG, and of oriLyt region were CGTCTTACTGCCCAGCCTACT and AGTGGGAGGGCAGGAAAT. Experiments examining EBNA1 binding to region III used the primer sets GACCACTGAGGGAGTGTTCCACAG and ACACCGTGCGAAAAGAAGCAC described in Yoshioka et al [36].

### EBNA1 ChIP assays performed on transfected plasmids

CNE2Z cells [70] were plated in 6 cm dishes and transfected with 50 pmols of siRNA against GFP or siRNA against USP7. siRNA transfections were repeated twice at 24 hour intervals for a total of 3 rounds of siRNA transfection over 72 hours. Cells were then moved to 10 cm dishes and transfected with 5 µg of pc3OriP, pc3OriPEBNA1 or pc3OriPΔ395–450 and 250 ng pLacZ plasmid containing LacZ cDNA. 24 hours post-transfection, cells were fixed with 1% formaldehyde, lysed in RIPA buffer (20 mM Tris pH 8.0, 150 mM NaCl, 1% NP40, 0.1% Sodium Deoxycholate, 1 mM PMSF) containing protease inhibitor cocktail (Sigma, P8340) and sonicated briefly to shear the DNA. Clarified lysates were precleared with Protein A/G beads (Santa Cruz, SC-2003) prior to immunoprecipitation with EBNA1 R4 antibody and normal rabbit IgG (Santa Cruz, SC-2345). Protein cross links were reversed in the immunoprecipitated DNA by incubating at 65°C for 16 hrs. DNA was purified using QIAquick Gel Extraction Kit (Qiagen, 28704) and analyzed by quantitative RT-PCR using LightyCycler 480 DNA SYBR Green I Master (Roche, 04707516001) and a Rotorgene Q-PCR system (Corbett Research). Primers used for DS are as described above. Primers used for FR and lacZ quantification were CCCGGATACAGATTAGGATAGC and TGTTGCCATGGGTAGCATA for FR and ATATTGAAACCCACGGCATGGTGC and TTTGATGGACCATTTCGGCACAGC for lacZ.

### Transcription activation assay

EBNA1 transactivation assays were performed as described previously [71] with the following modifications. CNE2Z cells were transfected with siRNA against GFP or USP7 as described above, then were moved to 10 cm dishes 24 hour prior to transfection with 2 µg of pFRTKCAT reporter construct (kindly provided by Bill Sugden) and 180 ng of pc3OriP or pc3Orip containing expression cassettes for EBNA1 [15] or EBNA1Δ395–450. 48 hrs later, cells were harvested and lysed using three rounds of freezing and thawing. 15 µg of total protein from each sample was assayed for chloramphenicol acetyltransferase activity using several reaction times and results from a point in the linear range was reported.
